# Occupational exposure to HIV and utilization of post-exposure prophylaxis among healthcare workers at St. Peter’s specialized hospital in Addis Ababa, Ethiopia

**DOI:** 10.1038/s41598-023-34250-4

**Published:** 2023-04-29

**Authors:** Dejen Tsega, Binyam Gintamo, Zelalem Negash Mekuria, Negesu Gizaw Demissie, Zemichael Gizaw

**Affiliations:** 1 Department of Public Health, Addis Ababa Medical and Business College, Addis Ababa, Ethiopia; 2 Yekatit 12 Medical College, Addis Ababa, Ethiopia; 3 Department of Medical Nursing, School of Nursing, College of Medicine and Health Sciences, University of Gondar, Gondar, Ethiopia; 4 Department of Environmental and Occupational Health and Safety, Institute of Public Health, College of Medicine and Health Sciences, University of Gondar, Gondar, Ethiopia

**Keywords:** Health care, Health occupations

## Abstract

Healthcare workers are susceptible to blood borne pathogens, such as human immunodeficiency virus (HIV). Occupational exposure to HIV infection among healthcare workers is becoming a global public health concern. However, there is limited evidence about occupational exposure of healthcare workers to HIV and utilization of post-exposure prophylaxis in Addis Ababa, Ethiopia. Accordingly, this study was conducted to assess the prevalence of occupational exposure to HIV and utilization of post exposure prophylaxis among healthcare workers at St. Peter’s specialized hospital, Addis Ababa, Ethiopia. A health facility-based cross-sectional study was conducted among 308 randomly selected healthcare workers in April 2022. Structured and pretested self-administered questioner was used to collect data. Occupational exposure to HIV was taken as any percutaneous injury or blood or other body fluids exposure while administering medications, specimen collection, and other procedures with HIV confirmed patients. Multivariable binary logistic regression analysis was used to identify factors associated with occupational exposure to HIV and utilization of post-exposure prophylaxis. Statistically significant association was declared on the basis of adjusted odds ratio with 95% confidence interval and p-value less than 0.05. The study found that 42.3% (95% CI 36.6, 47.9%) of the healthcare workers had occupational exposure to HIV during their career time, out of whom 16.1% (95% CI 11.9, 20.3%) used post-exposure prophylaxis. Healthcare workers with lower-level education such as diploma (AOR: 0.41, 95% CI 0.17, 0.96) and BSc (AOR: 0.51, 95% CI 0.26, 0.92), and healthcare workers who received infection prevention training (AOR: 0.55, 95% CI 0.33, 0.90) had less risk of exposure to HIV. On the other hand, nurses (AOR: 1.98, 95% CI 1.07, 3.67), midwifes (AOR: 3.79, 95% CI 1.21, 11.9), and physicians (AOR: 2.11, 95% CI 1.05, 4.22) had high risk of exposure to HIV compared with other professionals. Moreover, healthcare workers with BSc degree compared with healthcare workers with masters degree (AOR: 3.69, 95% CI 1.08, 12.6), healthcare workers with long service year (AOR: 3.75, 95% CI 1.64, 8.57), and healthcare workers who are working in facilities where prophylaxis is available (AOR: 3.41, 95% CI 1.47, 7.91) had higher odds to utilize post-exposure prophylaxis. Significant proportion of healthcare workers included in the current study had occupational exposure to HIV and very few of them used post-exposure prophylaxis. Healthcare workers need to use appropriate personal protective equipment, safely manage contaminated equipment, and safely administered medications and collect specimen to protect themselves from exposure to HIV. Moreover, use of post-exposure prophylaxis should be promoted when exposure exists.

## Introduction

Occupational exposure to infectious agents is one of the most important risk factors for HIV transmission among healthcare employees. Healthcare workers face a greater challenge as a result of their duties and responsibilities^1–3^. An occupational exposure that may place a healthcare workers at risk of HIV infection is defined as a percutaneous injury (e.g. a needle stick or cut with a sharp object), contact of mucous membrane or contact of skin (especially when the exposed skin is chapped, abraded or afflicted with dermatitis or when the contact is prolonged or involves an extensive area) with blood, tissues or other potentially infectious body fluids^4^. According to the World Health Organization (WHO), it is estimated that about 3 million HCWs are exposed to blood-borne pathogens each year and occupational exposure causes approximately 170,000 HIV infections^5^.

The risk of infections from blood-borne pathogens is increased by a number of circumstances, including absence of fundamental personal protection equipment, poor adherence to safety procedures, excessive use of injectable therapy, and needle-stick or sharp injuries^6–9^. The prevalence of HIV infection among patients, frequency of incidents in which HCWs are exposed to HIV-infected fluids, and the likelihood of transmission following occupational HIV exposure all affect the occupational risk of HIV infection among healthcare workers^1,10^. It is believed that 56.2% of healthcare workers worldwide sustained needle-stick and sharp injuries during the course of their careers, making needle-stick injuries the most common form of HIV exposure in healthcare settings^11^. However, there is a less than 1% chance of contracting HIV through a needlestick wound, and there is a less than 0.1% chance of getting exposed through direct skin contact with the fluid^12^.

There are many ways to prevent occupational exposure to HIV. Healthcare workers should assume all body fluids are infectious and take precautions such as use of protective covering like gloves and goggles, wash hands and other skin areas right after contact with blood and body fluids, careful handling and disposal of needles and sharp instruments, use of available safety devices to prevent needle stick injuries. If an exposure does occur, induce bleeding at the site of a skin puncture by applying gentle pressure around the wound, rinse the area well with water for a skin or mucous splash, know the infected person’s information, report to supervisor and coworkers, and seek immediate medical care^12–16^.

Post-exposure prophylaxis use is an important medical care to reduce the risk of HIV after occupational exposure occurred. Post-exposure prophylaxis provide 81% protection when started between 60 min and 72 h and followed for 28 days^17^. Post-exposure prophylaxis for HIV is the use of antiretroviral medications for a brief period of time to lower the risk of HIV infection following potential occupational or sexual exposure. Post-exposure prophylaxis for HIV should be administered as part of a comprehensive universal precaution package in the health sector^18^. Occupational exposure to HIV and not using post-exposure prophylaxis cause occupational burnout among healthcare workers due to severe adverse psychological pressure, such as stress and anxiety. This could largely erode the quality of healthcare services and in turn increase the risk of injuries to healthcare workers. The healthcare system needs therefore identify the predisposing factors for high risk of exposure. Accordingly, this study was conducted to assess the prevalence of occupational exposure to HIV, post exposure prophylaxis uses and associated factors among healthcare workers at St. Peter’s specialized hospital, Addis Ababa, Ethiopia.

## Methods

### Study design and setting

A health facility-based cross-sectional study was employed in St. Peter’s specialized hospital in Addis Ababa, Ethiopia. The hospital is located high in the Entoto mountain range, north of the city. The hospital was established in the era of Emperor Haile Selassie in 1948. Currently, the hospital has a total 936 healthcare workers [289 physicians, 343 nurses, 49 midwifes, 64 pharmacists, 68 laboratory personnels, 29 anesthetists, 39 public health officers, 25 radiographers, 9 dentists, 7 psychiatrists, 3 ophthalmologists, and 11 other healthcare workers (environmental health and optometrists)].

### Sample size calculation and sampling procedures

Sample size was calculated using single population proportion formula with the following assumptions: prevalence of occupational exposure to HIV in Dilla university referral hospital, southern Ethiopia = 76.1%^19^, 95% confidence level, 5% level of significance, 5% margin of error.

n = $\frac{{Z\alpha^{2} p\left( {1 - p} \right)}}{{d^{2} }}$ = $\frac{{1.96^{2} *0.761\left( {1 - 0.761} \right)}}{{0.05^{2} }}$ = 280. The final sample size became 308 after considering 10% non-response rate. All healthcare workers who had a potential to be exposed to HIV in their day-to-day professional activities were included in the study. Simple random sampling technique was used to recruit study subjects. We first prepared a sampling frame of eligible healthcare workers using their list obtained from the human resource department, and we then selected the study subjects using computer generated random number.

### Measurement of outcome variables

Occupational exposure to HIV, the primary outcome variable of this study was defined as any percutaneous injury and blood or other body fluids splash resulted while administering medications, specimen collection, and other procedures with HIV confirmed patients that may exposed healthcare workers to blood-borne pathogens^20^. Post-exposure prophylaxis use is the administration of antiretroviral medication within 72 h of contact with potentially contaminated blood or other body fluids in order to reduce the risk of infection^20^. For low-risk HIV infections, a combination of Tenofovir (TDF) + Lamivudine (3TC) or Zidovudine (AZT) + Lamivudine (3TC) while for high-risk exposures triple therapy should be used i.e., Zidovudine (AZT) + Lamivudine (3TC) + Lopinavir (LPV), Zidovudine (AZT) + Lamivudine (3TC) + Atazanavir (Atv), Tenofovir (TDF) + Lamivudine (3TC) + Dolutegravir (DTG), Tenofovir (TDF) + Lamivudine (3TC) + Lopinavir (LPV) and Tenofovir (TDF) + Lamivudine (3TC) + Atazanavir (Atv)^20^.

### Data collection procedures

Structured and pretested self-administered questionnaire was used to collect data. The questionnaire was adapted from published articles^20–24^. The questionnaire was initially prepared in English and translated into Amharic version, and back translated to English language to check for consistency. The questionnaire consists of socio demographic characteristics and behavioral factors, exposure to HIV, and post-exposure prophylaxis use. The data collection process was facilitated by three BSc nurses. Data were collected after obtaining written consent from the study participants. Data collection facilitators checked completeness of the questionnaire upon return.

### Data processing and analysis

Data were entered to Epi-data version 3.1 epidemiological software and exported to Statistical Package for Social Sciences (SPSS) version 25 for further analysis. For most variables, data were presented by frequencies and percentages. We included predictors to the multivariable binary logistic regression model based on bivariate *p* value (*p* < 0.25). Statistically significant associations in the multivariable model were identified on the basis of adjusted odds ratio (AOR) with 95% confidence interval (CI) and *p* values < 0.05. Model fitness was check using Hosmer and Lemeshow goodness-of-fit test.

### Ethics approval and consent to participate

Ethical clearance was obtained from the Institutional Review Board of Addis Ababa Medical and Business College (Reference number: AAMBC/STU/10,842/14) and submitted to St. Peter’s specialized hospital for permission. There were no risks due to participation and the collected data were used only for this research purpose with complete confidentiality. Written informed consent was obtained from the study participants. All the methods were carried out in accordance with relevant guidelines and regulations.

## Results

### Sociodemographic characteristics of study participants

From a total of 308 study participants, 298 of them returned the completed questioners with a response rate of 96.7%. The study participants were aged between 21 and 45 years and the mean (± SD) age was 30 (± 4.10) years. More than half, 158 (53%) of the study participants were married at the time of data collection and 151 (50.7%) of the study participants were Orthodox Christians by their religion. One hundred and nineteen (63.8%) of the study participants were male. Two-third, 198 (66.4%) of the study participants were Bachelor of Science degree holders and 108 (36.2%) of them were nurses. One hundred and fifty-three (51.3%) of the study participants had less than five years of work experience. One hundred and seventy-nine (60.1%) of the healthcare workers reported that they did not take on job infection prevention training (Table 1).

**Table 1:** Sociodemographic characteristic of healthcare workers (n = 298) in St. Peter’s specialized hospital in Addis Ababa, Ethiopia, April 2022.

Socio-demographic variables	Frequency	Percent (%)
Sex		
Male	190	63.8
Female	108	36.2
Age		
21–25 years	33	11.1
26–30 years	134	45.0
31–35 years	93	31.2
≥ 36 years	38	12.8
Marital status		
Single	140	47.0
Married	158	53.0
Religion		
Orthodox	151	50.7
Muslim	61	20.5
Protestant	52	17.4
Catholic	13	4.4
Other Christians	21	7.0
Profession		
Nurse	108	36.2
Midwife	16	5.4
Physician	68	22.8
Laboratory	22	7.4
Anesthetist	10	3.4
Health officer	13	4.4
Environmental health and optometrists	61	20.5
Educational status		
Diploma	49	16.4
BSc degree	198	66.4
MSc/MPH	40	13.4
Specialist	11	3.7
Work experience		
≤ 5 years	153	51.3
6–10 years	94	31.5
> 10 years	51	17.1
Working units		
Emergency	26	8.7
Pediatrics wards	26	8.7
Medical wards	34	11.4
Surgical wards	25	8.4
Operation room	32	10.7
ICU	24	8.1
OPD	28	9.4
MCH	24	8.1
Laboratory department	22	7.4
Psychiatric, toxicology, cardiac, and multi drug resistance units	57	19.1
Training on infection prevention		
Yes	119	39.9
No	179	60.1

*ICU* Intensive care units, *OPD* Outpatient department, *MCH* Maternal and child health.

### Occupational exposure of healthcare workers to HIV

One hundred and twenty-six (42.3%) (95% CI: 36.6, 47.9%) of the healthcare workers included in the current study had occupational exposure to HIV during their career time, out of which 27.8% exposed once, 28.6% exposed twice, 36.5% exposed three times, and 7.1% exposed four times and above. Forty-four (35%) of the healthcare workers reported that they exposed to HIV at workplace in the last three months prior to the survey. Fifty-two (42.9%) and 39 (31%) of the healthcare workers experienced needle-stick injuries and blood splash exposures, respectively. Eighty-four (66.7%) of the healthcare workers exposed to HIV while giving injections and 67 (53.2%) of the healthcare workers exposed during recapping needles. Eighty-two (65.1%) of the healthcare workers exposed to HIV during the day-time work shift. Fifty-nine (46.8%) of the healthcare workers did not use personal protective equipment during exposures (Table 2).

**Table 2:** Occupational exposure of healthcare workers (n = 126) to HIV in St. Peter’s specialized hospital in Addis Ababa, Ethiopia, April 2022.

Variables	Frequency	Percent (%)
Type of accident/exposure did you experience		
Needle stick injury	54	42.9
Blood splash	39	31.0
Mucous splash	7	5.6
Other body fluids	26	20.6
Activities during exposure		
Giving injections	84	66.7
Recapping needles	67	53.2
During surgery	36	28.6
Specimen collection	13	10.3
Collection of wastes	38	30.2
Number of exposures		
One time	35	27.8
Two times	36	28.6
Three times	46	36.5
≥ Four time	9	7.1
When was your last exposure?		
In the previous 3 months	44	35.0
In the previous 6 months	40	31.7
Before the previous 6 months	42	33.3
Use of personal protective equipment at time of exposure		
Yes	67	53.2
No	59	46.8
Which PPE were you using (n = 67)		
Glove	67	100.0
Mask	45	67.7
Apron	16	23.9
Goggle	11	16.4
Gown	45	67.2
Other	13	19.4
Reason/s for not using personal protective equipment (n = 59)		
Equipment not available	35	59.3
Negligence and being hurry	24	40.7
Working shift at exposure time		
Day	82	65.1
Night	44	34.9
Did you report the accident?		
Yes	77	61.1
No	49	38.9

### Utilization of post exposure prophylaxis

Forty-eight (16.1%) (95% CI 11.9–20.3) of the healthcare workers used post-exposure prophylaxis. One hundred and sixty-seven (56.0%) of the healthcare workers reported that they did not take training on post-exposure prophylaxis and 99 (33.2%) of the healthcare workers reported that post-exposure prophylaxis is not available in their facility (Table 3).

**Table 3:** Utilization of post-exposure prophylaxis among healthcare workers (n = 298) who had occupational exposures to HIV in St. Peter’s specialized hospital, Addis Ababa, Ethiopia, April 2022.

Variables	Frequency	Percent (%)
Training on post-exposure prophylaxis		
Yes	131	44.0
No	167	56.0
Post-exposure prophylaxis available in this facility		
Yes	199	66.8
No	99	33.2
Have you ever used post-exposure prophylaxis		
Yes	48	16.1
No	250	83.9
When you started prophylaxis after exposure? (n = 48)		
Within 24 h	30	62.5
After 48 h	16	33.3
Within 72 h	2	4.2

### Factors associated with occupational exposure to HIV

Sex, age, marital status, field of study, educational status, work experience, infection prevention training, and availability of post-exposure prophylaxis in the facility were the candidate variables for the multivariable model and in the adjusted model, occupational exposure to HIV was significantly associated with educational status, field of study, and infection prevention training. Healthcare workers who were diploma holders had 59% less risk of exposure to HIV at the workplace compared with healthcare workers who had masters degree in their field of study (AOR: 0.41, 95% CI 0.17, 0.96). Healthcare workers who received infection prevention training had 45% lower odds of occupational exposure to HIV risky conditions compared with healthcare workers who did not receive infection prevention training (AOR: 0.55, 95% CI 0.33, 0.90). Moreover, the odds of having occupational exposure to HIV risky conditions among nurses were 1.98 times higher compared with other healthcare workers (AOR: 1.98, 95% CI 1.07, 3.67) (Table 4).

**Table 4:** Factors associated with occupational exposure to HIV among healthcare workers at St. Peter’s specialized hospital in Addis Ababa, Ethiopia, April 2022.

variable	Occupational exposure to HIV		COR (95%CI)	AOR with 95% CI
	Yes	No		
Sex				
Male	74	116	0.68 (0.42, 1.10)	0.96 (0.45, 1.29)
Female	52	56	1.0	1.0
Age				
21–25 years	10	23	0.35 (0.13, 0.93)	0.42 (0.10, 1.80)
26-30 years	51	83	0.49 (0.24, 1.03)	0.58 (0.18, 1.89)
31–35 years	44	49	0.72 (0.34, 1.55)	0.95 (0.35, 2.57)
≥ 36 Years	21	17	1.0	1.0
Marital status				
Unmarried	51	89	1.57 (0.99, 2.51)	0.96 (0.54, 1.71)
Married	75	83	1.0	1.0
Professions				
Nurse	50	58	2.03 (1.11, 3.71)	1.98 (1.07, 3.67)*
Midwifery	10	6	3.93 (1.29, 11.9)	3.79 (1.21, 11.90)*
Laboratory	8	14	1.34 (0.50, 3.61)	1.04 (0.37, 2.89)
Physician	33	35	2.22 (1.14, 4.33)	2.11 (1.05, 4.22)*
Other professionals	25	59	1.0	1.0
Educational status				
Diploma	16	33	0.36 (0.16, 0.83)	0.41 (0.17, 0.96)*
Degree	81	117	0.52 (0.28, 0.97)	0.51 (0.26, 0.92)*
Second degree and above	29	22	1.0	1.0
Work experience				
≤ 5 years	60	93	0.62 (0.32, 1.12)	1.25 (0.42, 3.67)
6–10 years	40	54	0.71 (0.35, 1.41)	0.77 (0.30, 2.00)
> 10 years	26	25	1.0	1.0
Training on infection prevention				
Yes	41	78	0.58 (0.36, 0.93)	0.55 (0.33, 0.90)**
No	85	94	1.0	1.0
Availability of post-exposure prophylaxis				
Yes	92	107	1.64 (0.99, 2.70)	1.51 (0.89, 2.54)
No	34	65		

*Statistically significant at *p* < 0.05, **statistically significant at *p* < 0.01.

*AOR* adjusted odds ratio, *HIV* Human immunodeficiency virus, *CI*: Confidence interval, Hosmer and Lemeshow test = 0.147, other professional: public health officer, anesthetists, psychiatry, radiographers, pharmacy personnels, and optometry.

### Factors associated with utilization of post-exposure prophylaxis

Age of respondents, marital status, field of study, educational status, work experience, having training on infection prevention, and availability of post-exposure prophylaxis were the candidate variables for the multivariable binary logistic regression analysis. In the adjusted model, educational status, work experience, and availability of post-exposure prophylaxis in the facility were significantly associated with utilization of post-exposure prophylaxis. The odds of utilization of post-exposure prophylaxis was 3.69 times higher among healthcare workers who had master’s degree and above compared with diploma holders (AOR: 3.69, 95% CI: 1.08, 12.6). Healthcare workers who had long work experience had higher odds to use post-exposure prophylaxis (AOR: 3.75, 95% CI: 1.64, 8.57). Moreover, the odds of utilization of post-exposure prophylaxis was 3.41 times higher among healthcare workers who reported that post-exposure prophylaxis is available in their facility (AOR: 3.41, 95% CI 1.47, 7.91) (Table 5).

**Table 5:** Factors associated with utilization of post-exposure prophylaxis among healthcare workers at St. Peter’s specialized hospital in Addis Ababa, Ethiopia, April 2022.

Variable	Post-exposure prophylaxis use		COR with 95% CI	AOR with 95% C
	Yes	No		
Age				
21–25 years	5	28	0.57 (0.17, 1.93)	5.47 (0.84, 35.3)
26-30 years	14	120	0.37 (0.14, 0.95)	1.88 (0.44, 7.98)
31–35 years	20	73	0.88 (0.36, 2.16)	2.07 (0.66, 6.48)
≥ 36 Years	9	29	1.0	1.0
Marital status				
Unmarried	16	124	1.96 (1.02, 3.76)	0.71 (0.31, 1.62)
Married	32	126	1.0	1.0
Profession				
Nurse	16	92	1.28 (0.55, 3.00)	1.06 (0.43, 2.61)
Midwifery	4	12	1.26 (0.66, 9.14)	1.43 (0.34, 5.97)
Laboratory	4	18	1.64 (0.46, 5.84)	0.98 (0.24, 3.91)
Physician	14	54	1.91 (0.79, 4.64)	1.37 (0.51, 3.69)
Others	10	74	1.0	1.0
Educational status				
Diploma	4	45	0.19 (0.06, 0.63)	1.77 (0.58, 5.41)
Degree	28	170	0.36 (0.17, 0.73)	3.69 (1.08, 12.6)*
Second degree and above	16	35	1.0	1.0
Work experience				
≤ 5 years	14	139	1.0	1.0
6–10 years	20	74	2.68 (1.28, 5.60)	2.13 (0.99, 4.57)
> 10 years	14	37	3.75 (1.64, 8.57)	2.72 (1.12, 6.59)*
Training on infection prevention				
Yes	15	104	0.68 (0.33, 1.23)	0.66 (0.33, 1.32)
No	33	146	1.0	1.0
Availability of prophylaxis				
Yes	41	158	3.41 (1.47, 7.91)	3.09 (1.30, 7.33)**
No	7	92	1.0	1.0

*Statistically significant at *p* < 0.05, ** statistically significant at *p* < 0.01.

*AOR* adjusted odds ratio, *CI* Confidence interval Hosmer and Lemeshow test = 0.285, other professional = public health officer, anesthetists, psychiatry, radiographer, pharmacypersonnels, and optometry.

### Discussion

This hospital-based cross-sectional study was conducted to assess occupational exposure of healthcare workers to HIV and post-exposure prophylaxis use in St. Peter’s specialized hospital, Addis Ababa, Ethiopia and found that 42.3% (95% CI 36.64, 47.92%) of the healthcare workers exposed to HIV risky conditions during their career time and 16.1% (95% CI 11.9–20.3) of the exposed healthcare workers used post-exposure prophylaxis. The prevalence of occupational exposure to HIV in the current study is comparable with findings of studies in Gondar city, northwest Ethiopia (40.4%)^25^ and Nigeria (45.0%)^26^. The prevalence of occupational exposure to HIV in the current study is also lower than studies in Bule Hora General Hospital, Ethiopia (61.6%)^20^ and Tanzania (50.6%)^27^. On the other hand, this study finding is higher than studies in South Africa (10.6%)^28^ and Tanzania (35.1%)^22^. This high prevalence of occupational exposure to HIV risky conditions might be explained by poor safety system that includes lack of basic personal protective equipment, poor adherence to safety practices, and poor sharp waste management in the healthcare facilities. Furthermore, overuse of injectable therapy, suturing, recapping needles, bend or break needles, removing needles from syringes after injection, washing contaminated instruments, workload, working hastily, fatigue, crowded work environment may associate with needle-stick and sharp injuries that may result exposure to HIV. Moreover, 16.1% (95% CI 11.9, 20.3%) of the healthcare workers who had occupational exposure to HIV risky conditions used post-exposure prophylaxis, which is in agreement with studies in Ethiopia (19.6%)^29^, Cameron (18.9%)^17^, and Tanzania (16.7%)^27^. However, this study finding is lower than findings of studies in west Guji zone of Ethiopia (24.3%)^20^, South Africa (58.8%)^28^, and Tanzania (26.4%)^27^. This low-level utilization of post-exposure prophylaxis might be explained by frequent stock-outs and continuous absence of post-exposure prophylaxis. In addition, some individuals who exposed to HIV risky conditions might perceived that their risks to HIV due to occupational exposure might be low.

This study revealed that occupational exposure to HIV risky conditions was associated with educational status. Healthcare workers with lower education level had lower odds of occupational exposure to HIV risky conditions. This might be due to healthcare workers who have higher educational status performing advanced surgical procedures which might increase their exposure to HIV risky conditions^30^.

In the current study, occupational exposure to HIV risky conditions was significantly associated with infection prevention training. Healthcare workers who received infection prevention training had lower risks of occupational exposure to HIV. This study finding is in line with findings of other studies^25,31^. This might be due to the fact that infection prevention training is an effective strategy to develop knowledge and skills on safety measures and trained healthcare workers can protect themselves and other coworkers from work place injuries^32^.

This study found that occupational exposure to HIV risky conditions was statistically associated with field of study. For instance, nurses, midwifes, and physicians had higher odds of exposures compared with other healthcare workers such as public health officers, anesthetists, psychiatrists, radiologists, pharmacists, and optometrists). This finding is in agreement with a study in Serbia^33^. The possible explanations could be nurses, midwifes, and physicians had more frequent contact with patients during healthcare provision as well as they do invasive procedure like injection of medication, surgery, and delivery that increases their risk to healthcare associated infections^34^.

Furthermore, this study depicted that utilization of post-exposure prophylaxis among healthcare workers exposed to HIV risky conditions was significantly associated with educational status. Healthcare workers who had master’s degree and above had higher odds of post-exposure prophylaxis use, which is in agreement with a study in Uganda^35^. This could be due to the fact that healthcare workers at a higher educational level may have awareness about the mechanisms how post-exposure prophylaxis helps to prevent from HIV infection so that they may not have fear of side effects and could develop positive attitude. Moreover, healthcare workers at a higher educational level can identify risky conditions for acquiring HIV infection^36^.

The current study reported that utilization of post-exposure prophylaxis was associated with work experience. Healthcare workers who had long work experience had higher odds to use post-exposure prophylaxis. This finding is in line with a study in Uganda^35^. This might be due to the fact that experienced healthcare workers might have information about advantage of post-exposure prophylaxis use over its side effects^37^**.** Moreover, as service year increases, the perceived vulnerability to infection might be high that may motivate healthcare workers to use prophylaxis when they are exposed to HIV risky conditions and long work experience might be associated with increased adherence to infection prevention strategies^38^.

This study also revealed that availability of post-exposure prophylaxis in the facility at the time when healthcare workers were exposed to HIV risky conditions was associated with higher odds of post-exposure prophylaxis utilization. The continuous availability of essential medicines within healthcare facilities plays an important role in promoting utilization of health services. On the other hand, frequent stock-outs of medicines have been shown to influence healthcare utilization. The continued absence of medicines in health facilities influences healthcare utilization and individual decisions^39–41^.

As a limitation, the self-reported data may not be reliable since the study subjects may make the more socially acceptable answer rather than being truthful and they may not be able to assess themselves accurately. The study might be also affected by recall bias since we asked healthcare workers to recall occupational exposures during their career time. There might be also possibility of unmeasured confounders (e.g., medical conditions). Moreover, the variable of interest was not equally important for all departments. It might be more in some departments such as laboratory, emergency, delivery, etc. However, we didn’t address this variation in the analysis. The number of healthcare workers in each profession was not also proportional, even if random sampling was utilized. This study also included only one hospital data in Ethiopia. All these may affect the generalizability of study results.

## Conclusion

Significant proportion of healthcare workers included in the current study had occupational exposure to HIV and very few of them used post-exposure prophylaxis. This implies that occupational exposure to HIV in the studied healthcare facility is a great concern that may result exposure of healthcare workers to blood-borne pathogens. Healthcare workers need, therefore, use personal protective equipment (includes gloves, gowns, masks, and eye protection), safely manage contaminated equipment and other items in the patient environment, and follow safety procedures during medication administration, specimen collection, and other procedures to protect themselves from HIV and other blood-borne pathogens including use of post-exposure prophylaxis when needed. Moreover, the health facility needs to strengthen the health and safety culture of the institution and availability of post-exposure prophylaxis.

## Data Availability

Data will be made available upon requesting DT, who is the primary author of this study.
